# The NLRP3 inflammasome is a potential mechanism and therapeutic target for perioperative neurocognitive disorders

**DOI:** 10.3389/fnagi.2022.1072003

**Published:** 2023-01-04

**Authors:** Jiayue Li, Li Li, Jiannan He, Jianhong Xu, Fangping Bao

**Affiliations:** ^1^Department of Anesthesiology, The Fourth Affiliated Hospital, International Institutes of Medicine, Zhejiang University School of Medicine, Yiwu, China; ^2^Department of Anesthesiology, The First Affiliated Hospital Zhejiang University School of Medicine, Hangzhou, China

**Keywords:** perioperative neurocognitive disorders, pyroptosis, NLRP3 inflammasomes, posttranscriptional modifications, targeted protein degradation, nanotechnology

## Abstract

Perioperative neurocognitive disorders (PNDs) are frequent complications associated with cognitive impairment during the perioperative period, including acute postoperative delirium and long-lasting postoperative cognitive dysfunction. There are some risk factors for PNDs, such as age, surgical trauma, anesthetics, and the health of the patient, but the underlying mechanism has not been fully elucidated. Pyroptosis is a form of programmed cell death that is mediated by the gasdermin protein and is involved in cognitive dysfunction disorders. The canonical pathway induced by nucleotide oligomerization domain (NOD)-, leucine-rich repeat (LRR)- and pyrin domain-containing protein 3 (NLRP3) inflammasomes contributes to PNDs, which suggests that targeting NLRP3 inflammasomes may be an effective strategy for the treatment of PNDs. Therefore, inhibiting upstream activators and blocking the assembly of the NLRP3 inflammasome may attenuate PNDs. The present review summarizes recent studies and systematically describes the pathogenesis of NLRP3 activation and regulation and potential therapeutics targeting NLRP3 inflammasomes in PNDs patients.

## Introduction

Perioperative neurocognitive disorders (PNDs) are frequent complications associated with cognitive impairment during the perioperative period, including acute postoperative delirium (PD) and long-lasting postoperative cognitive dysfunction (POCD), which were once considered two distinguishing (Evered et al., 2018). Some PNDs patients develop Alzheimer’s disease (AD; Vanderweyde et al., 2010), and preclinical AD patients tend to suffer from PNDs when exposed to anesthesia and surgery (Evered et al., 2016). There are several risk factors for PNDs, including increasing age, surgical trauma, anesthetics, poor health, lower education levels, pain, and some preconditions, such as anticholinergic medications, mental disease, and abnormal sleep rhythm(Kalisvaart et al., 2006; Kotekar et al., 2018; Ni et al., 2019; Wei et al., 2019). Among the potential risk factors, advanced age is a relatively definite risk factor. With the aging of the population and the increased demand for surgery, it is foreseeable that the incidence of PNDs will increase (Wei et al., 2019) and lead to increased morbidity, mortality, and a heavier social burden (Androsova et al., 2015). Therefore, examination of PNDs pathogenesis and effective treatment strategies are important.

The underlying mechanism of PNDs has not been fully elucidated. Many recent clinical trials and experiments have proposed several hypotheses, including neuroinflammation (Cibelli et al., 2010; Safavynia and Goldstein, 2018; Xiang et al., 2019), synapse dysfunction (Xu et al., 2017), amyloid beta (Aβ) accumulation, and tau protein phosphorylation (Yu et al., 2020), which indicate that a common pathogenic pathway exists between PNDs and neurodegenerative disorders, especially AD. Among these, neuroinflammation may be the highlighted mechanism of PNDs (Cibelli et al., 2010; Safavynia and Goldstein, 2018; Xiang et al., 2019). This theory includes three parts: peripheral inflammation, central inflammation, and the link between these conditions. The elevation of proinflammatory cytokines in the periphery and cerebrospinal fluid is positively related to PNDs (Liu et al., 2018). Patients with high interleukin (IL)-1β in presurgical cerebrospinal fluid (CSF) tend to suffer from PNDs (Ji et al., 2013). Damaged peripheral cells caused by surgery release high molecular group box 1 protein (HMGB1), which is a type of damage-associated molecular pattern (DAMP) that may be sensed by Toll-like receptors (TLRs; Lotze and Tracey, 2005). HMGB1 is also increased in the hippocampus after surgery and may contribute to changes in the blood–brain barrier (BBB; He et al., 2012). The nuclear factor (NF)-κB signaling pathway is triggered and upregulates the release of cytokines [(TNF)-α, IL-1β, and IL-6], which maintain peripheral inflammation (Li et al., 2022). Changes in the permeability or integrity of the BBB may be the bridge between peripheral inflammation and neuroinflammation (Li et al., 2022). Notably, intestinal inflammation induces neuroinflammation *via* a certain mechanism and ultimately contributes to cognitive impairment (He et al., 2021). The imbalance of gut microbiota caused by surgery or anesthesia contributes to the development of PNDs (Lian et al., 2021).

Pyroptosis is an inflammatory form of programmed cell death, that leads to plasma membrane disruption, potassium efflux, and IL-1β and IL-18 release by canonical or noncanonical pathways. The NOD-, LRR-, and pyrin domain-containing protein 3 (NLRP3) inflammasome plays an important role in the canonical pyroptosis pathway, which is associated with neurodegenerative diseases, such as AD, Parkinson’s disease, and epilepsy (Moujalled et al., 2021; Xia et al., 2021), and is a pivotal role in PNDs (Zhang et al., 2021b). In recent studies, NLRP3 is significantly increased in the PNDs mouse model (Zhang et al., 2021b) and is associated with isoflurane-induced cognitive impairment (Wang et al., 2018; Yin et al., 2018). Inhibiting NLRP3 and caspase-1 (its downstream target) with MCC950 and AC-YVAD-CMK, respectively, reduced the expression of IL-1β and attenuated PNDs (Fan et al., 2018; Fu et al., 2020; Zhang Z. et al., 2021). It seems that NLRP3 could lead PNDs directly.

At the same time, NLRP3 has a strong relationship with the risk factors for PNDs. NLRP3 inflammasome increases in the hippocampus of aged rats (Wang et al., 2022). Pain contributes to PNDs (Chi et al., 2013), the NLRP3 inflammasome mediates postoperative mechanical pain, and knockout of NLRP3 reduced mechanical hypersensitivity and pain-like behavior in mice (Cowie et al., 2019). The NLRP3 inflammasome is also involved in abnormal sleep rhythm. The expression of the NLRP3 inflammasome (NLRP3, ASC, and active caspase-1) increased in the hippocampal CA1 region of mice in the sleep deprivation group, and this effect could be reversed by sleep recovery (Fan et al., 2021). NLRP3 inflammasome activation could be induced by Aβ accumulation and lead to Tau hyperphosphorylation, which may verify the role of the NLRP3 inflammasome in AD pathogenesis (Ising et al., 2019). In addition, colitis could upregulate neuroinflammation, Aβ deposition, and cognitive impairment, but this effect could be mitigated by kickout of NLRP3 (He et al., 2021).

Therefore, NLRP3 not only induces PNDs directly but also promotes risk factors for PNDs. The NLRP3 inflammasome plays a potential role in the pathogenesis of PNDs, and any treatment inhibiting the NLRP3 inflammasome pathway may be an effective strategy to treat PNDs. The present review summarizes recent studies and systematically describes the pathogenesis of NLRP3 activation and regulation and potential therapeutics targeting NLRP3 inflammasomes in PNDs patients.

## Components of NLRP3 and its mechanism

The NLRP3 inflammasome is a multiprotein complex that includes the NLRP3 protein, apoptosis-associated speck-like protein containing a CARD (ASC), and pro-caspase-1. The NLRP3 protein (the sensor of the NLRP3 inflammasome) is a pattern recognition receptor (PRR) that consists of an amino-terminal pyrin domain (PYD), a central NACHT domain (which has ATPase activity), and a carboxy-terminal LRR domain. ASC consists of an N-terminal PYD and a C-terminal caspase-recruitment domain (CARD), and pro-caspase-1 has an N-terminal CARD. After recognizing the stimulus, NLRP3 oligomerizes and recruits ASC *via* a PYD domain interaction, and then ASC recruits pro-caspase-1 *via* a CARD domain interaction and induces the self-cleavage of pro-caspase-1 (Swanson et al., 2019). The active form of caspase cleaves pro-IL-1β and pro-IL-18 into their mature forms (Lawlor and Vince, 2014). This step is the assembly process and effect of the NLRP3 inflammasome. There are potential therapeutic strategies for each step in this process.

**Figure 1:**
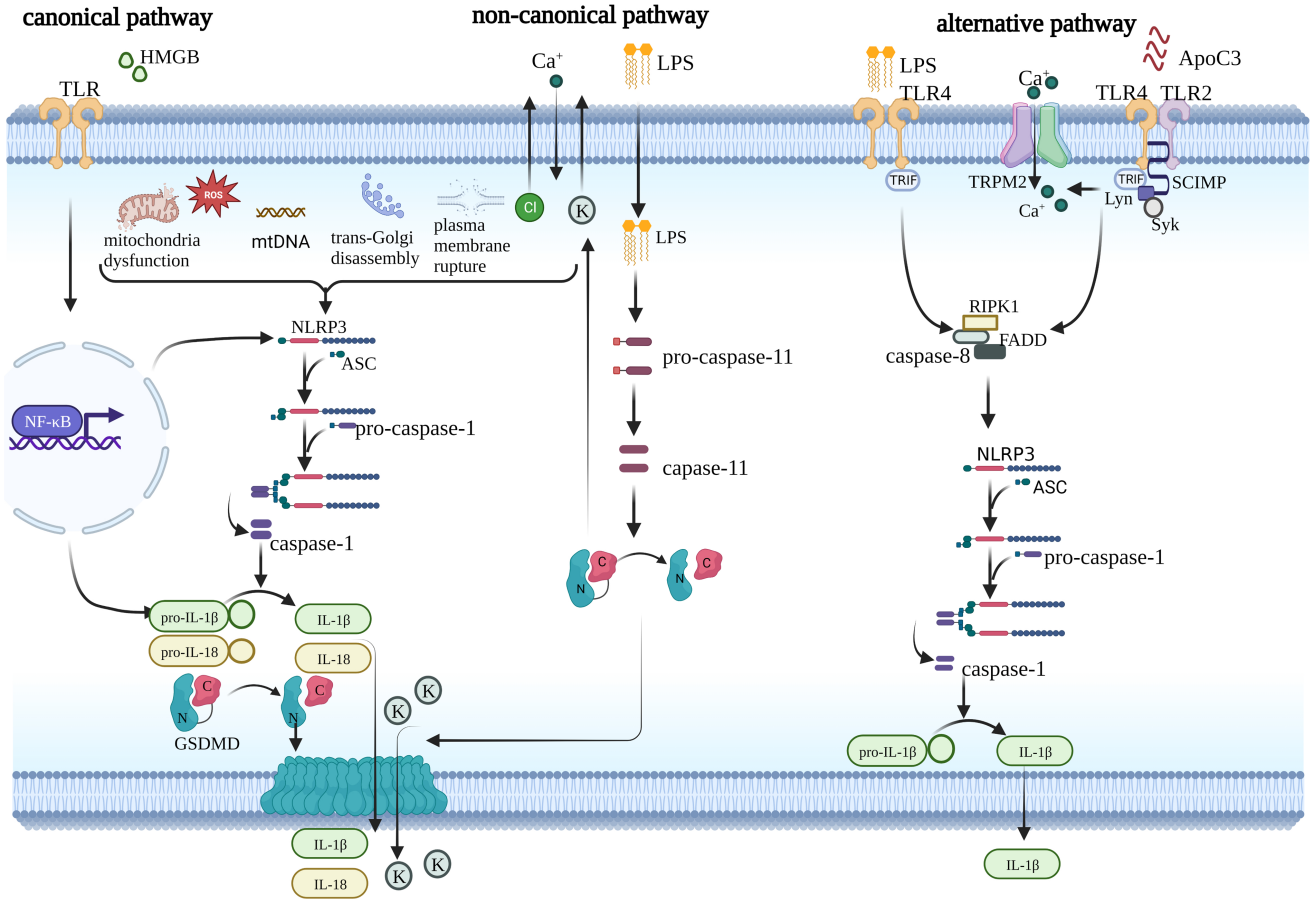
Three pathways of NLRP3 inflammasome activation. The canonical pathway requires two steps to complete: the priming step and the activation step. During the priming step, TLR or cytokine receptors sense stimuli and trigger the NF-κB pathway, which prompts the transcription of NLRP3 and pro-IL-1β and triggers numerous events upstream of the NLRP3 inflammasome, such as mitochondrial dysfunction, mitochondrial DNA (mtDNA) synthesis, reactive oxygen species (ROS), trans-Golgi disassembly, plasma membrane rupture, and ionic flux. Finally, GSDMD is induced into its active form, which creates pores in the plasma membrane and facilitates cytokine release. Noncanonical signaling is activated by LPS and mediated by caspase11 (caspase-4/5 in humans), which also independently induces pyroptosis and activates the canonical pathway via ionic flux. The alternative pathway does not induce pyroptosis. It is mediated by the LPS-induced TLR4-TRIF-RIPK1-FADD-CASP8 signaling pathway or ApoC3-induced TLR2/4-SCIMP-Lyn-Syk-TRPM2-CASP8 pathway. Caspase-8 promotes the assembly of the NLRP3 inflammasome and cytokine release.

There are three kinds of NLRP3 inflammasome activation pathways: the canonical, noncanonical, and alternative NLRP3 inflammasome pathways, as shown in Figure 1. The canonical pathway is also known as the two-signal model (Huang et al., 2021). The first signal from TLRs or cytokine receptors induces NLRP3 and pro-IL-1β expression *via* NF-κB activation and is the priming of the NLRP3 inflammasome. The second signal is triggered by microbial products or danger signals, such as ATP, pore-forming toxins, and viral RNA (Bauernfeind et al., 2009; He et al., 2016; Xing et al., 2017). Once these stimuli are sensed, the assembly and activation of the NLRP3 inflammasome is initiated as described above. Several events are regarded as activation signals of the NLRP3 inflammasome, including mitochondrial dysfunction (Zuo et al., 2020), mitochondrial DNA (mtDNA) synthesis (Zhong et al., 2018), reactive oxygen species (ROS) (Wei et al., 2019), ionic flux [potassium efflux (Muñoz-Planillo et al., 2013), calcium influx (Murakami et al., 2012), and chloride efflux (Tang et al., 2017; Swanson et al., 2019)], trans-Golgi disassembly (Gaidt et al., 2016), and plasma membrane rupture (Beckwith et al., 2020). In contrast to the canonical pathway, the noncanonical pathway is dependent on caspase-4/5 (mouse caspase-11), which may be converted to an active state *via* direct binding to LPS and lipid A. Active caspase-4/5/11 also induces pyroptosis *via* pore formation, which is characteristic of GSDMD (Shi et al., 2014). Plasma membrane rupture and K^+^ efflux further trigger activation of the NLRP3 inflammasome. The alternative pathway is completed by TLR4-TRIF-RIPK1-FADD-CASP8 signaling, which responds to LPS and ultimately causes IL-1β release (Gaidt et al., 2016). Apolipoprotein C3 (ApoC3) primes the alternative pathway *via* TLR2/4-SCIMP-Lyn-Syk-TRPM2-CASP8 in human monocytes. However, alternative NLRP3 inflammasome activation does not induce ASC speck formation or pyroptosis (Zewinger et al., 2020). Therefore, canonical and noncanonical NLRP3 inflammasome activation are described in this article because they induce GSDMD to execute pyroptosis.

GSDMD is the substrate of caspase-1/4/5/11 and may be cleaved to form the gasdermin-N-terminal and gasdermin-C-terminal. The latter autoinhibits the gasdermin-N-terminal, which is the functional segment that contributes to pyroptosis (Shi et al., 2017). Once the interaction of these two segments is disrupted by caspase, the GSDMs are activated (Shi et al., 2015). Therefore, there are two ways in which GSDMs are activated. First, proteases cleave and release the NT active domain, and second, the CT domain is mutated, which diminishes the negative regulation of the NT domain by the CT domain (Liu X. et al., 2021). Once the GSDMD-N-terminal is released, it oligomerizes in the membrane to form pores that permit the release of cytokines, such as IL-1β, and lead to ion flux, such as calcium and potassium, which may further prompt activation of the NLRP3 inflammasome (Liu et al., 2016; Fischer et al., 2021). GSDMD-induced pyroptosis contributes to cognitive disorders caused by sevoflurane neurotoxicity (Wen-Yuan et al., 2022). An increasing number of studies have shown that other members of the gasdermin family (GSDMA3, GSDMB (Zhou et al., 2020), GSDMC (Hou et al., 2020), and GSDME (Rogers et al., 2017; Wang Y. et al., 2017) contribute to pyroptosis *via* other caspases or molecules (Huang et al., 2021).

Based on the compound of NLRP3 and its mechanism, any blocking the assembly process of the NLRP3 inflammasome, inhibiting the activation signals of the NLRP3 inflammasome, and decreasing and dysfunction of GSDMD (downstream of NLRP3 inflammasome) could relieve PNDs.

## Posttranscriptional modifications (PTMs)

**Figure 2:**
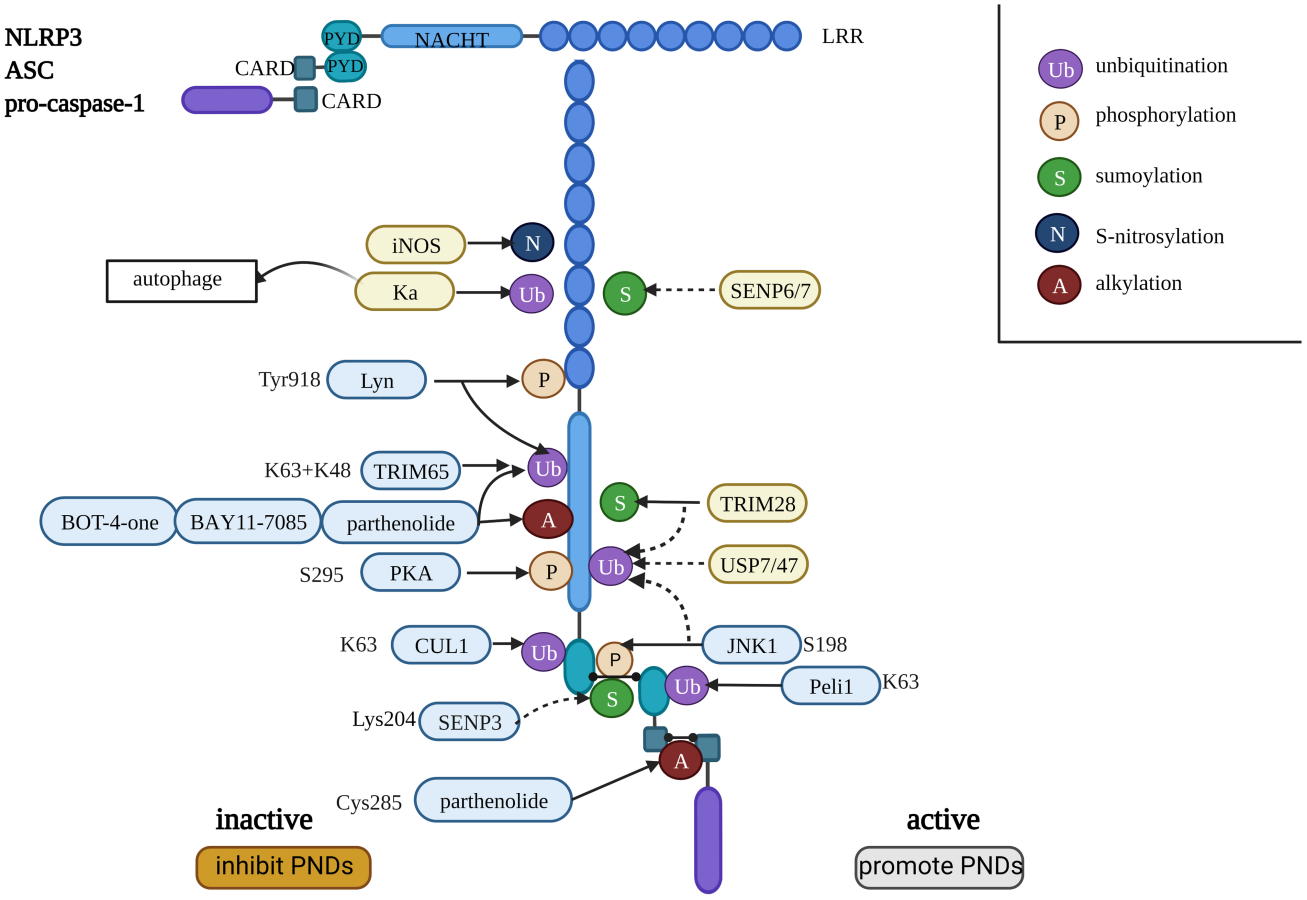
The consistency of the NLRP3 inflammasome and the regulation of PTMs on the NLRP3 inflammasome. The dotted line indicates the removal of small molecular modifications, and the solid line indicates the promotion of PTMs. The blue background indicates an explicit interaction site with NLRP3, and the yellow background indicates that the site is not clear. The molecules listed on the left side inactivate the NLRP3 inflammasome, and the factors on the right side activate the NLRP3 inflammasome.

PTM of the NLRP3 inflammasome, including ubiquitination, phosphorylation, small ubiquitin-like modifier (SUMO) ylation, alkylation, and S-nitrosylation, is involved in regulating inflammasome activation (Tang T. et al., 2021). PTMs may occur at any step in the pyroptosis pathway of proteins, such as NLRP3 and gasdermins (Fischer et al., 2021). Different PTMs do not work independently but interact with each other. The mechanism is very complex. We used the NLRP3 inflammasome as an example to briefly introduce the effects of PTMs, as shown in Figure 2. The molecule that promotes the activation of NLRP3 could induce PNDs, while the other inactive NLRP3 may be a potential treatment strategy for PNDs.

### Ubiquitination

Ubiquitination of a protein requires E1 (ubiquitin-activating enzyme), E2 (ubiquitin-conjugating enzyme), and E3 (ubiquitin ligase; Glickman and Ciechanover, 2002). E3 ubiquitin ligases are associated with the regulation of NLRP3 inflammasome activation by targeting NLRP3 inflammasome components (Lopez-Castejon, 2020). Several E3 ligases are associated with NLRP3 inflammasome activation (Akther et al., 2021). Wan et al. found that the core component of E3 ligases, cullin1 (CUL1), catalyzed NLRP3 ubiquitination to suppress the activation of NLRP3 in resting cells, and it disassociated from NLRP3 to facilitate the assembly and activation of the NLRP3 inflammasome to protect against inflammatory stimuli and infection (Wan et al., 2019). In contrast, Peli1 catalyzes K (lys)63 ubiquitination of ASC, which contributes to NLRP3 inflammasome activation by promoting ASC/NLRP3 interaction (Zhang et al., 2021a). Kaempferol (Ka) inhibits NLRP3 inflammasome activation by promoting autophagic degradation of the NLRP3 inflammasome and NLRP3 ubiquitination (Han et al., 2019). Deubiquitinases (DUBs) are important components of the ubiquitin system that counter the role of ubiquitinases to achieve balance. The DUBs USP7 and USP47 activate the NLRP3 inflammasome, but the specific mechanism is not clear (Palazón-Riquelme et al., 2018).

There are three mechanisms by which E3 ubiquitin ligases affect NLRP3 inflammasome activation, including lys48-, lys63- or mixed lys48- and lys63-linked ubiquitination. There are three functional mechanisms: proteasomal degradation of NLRP3, autophagic degradation of NLRP3, and NLRP3 inactivation without protein degradation (Tang T. et al., 2021). A recent study demonstrated that the E3 ubiquitin ligase TRIM65 was a negative regulator of NLRP3 inflammasome activation, and it suppressed the assembly and activation of the NLRP3 inflammasome by promoting K48 and K63 ubiquitination of NLRP3, which did not lead to its degradation (Tang T. et al., 2021).

### Phosphorylation

Phosphorylation mediates the priming, assembly, localization, and degradation of the NLRP3 inflammasome by regulating deubiquitination or ubiquitination of NLRP3 in macrophages (Gong et al., 2018). The phosphorylation of NLRP3 at S198 (mouse NLRP3 S194) by JNK1, a TLR-IRAK1/4 downstream kinase, is a key regulator of the deubiquitination and activation of NLRP3 (Song et al., 2017). The dephosphorylation of S198 or phosphorylation of the S3 residue hinders the homo-oligomerization of NLRP3 and its interaction with ASC to inhibit its activation (Mangan et al., 2018). Protein kinase A (PKA) promotes NLRP3 ubiquitination and deactivates ATPase by directly phosphorylating NLRP3 at S295 (mouse NLRP3 S291), which inhibits the activation of NLRP3 (Guo et al., 2016; Mortimer et al., 2016). The protein tyrosine kinase (PTK) Lyn phosphorylates NLRP3 at Tyr918 (mouse NLRP3 Y915) and promotes NLRP3 ubiquitination to inhibit inflammasome activation (Tang J. et al., 2021). Some kinases or phosphatases that promote NLRP3 inflammasome activation, such as protein tyrosine phosphatase nonreceptor 22 (PTPN22), never in mitosis A-related kinase 7 (NEK7), death-associated protein kinase (DAPK), and Bruton’s tyrosine kinase (BTK; Gong et al., 2018), may also be potential treatment targets.

Current clinical trials of NLRP3-targeting kinase inhibitors primarily focus on tumors (such as mantle cell lymphoma) and autoimmune diseases (such as rheumatoid arthritis), and whether NLRP3 phosphorylation is a practical treatment target needs further study (Gong et al., 2018).

### SUMO modification

SUMOylation works like ubiquitination, and its function relies on E1 activating enzymes, E2 conjugating enzymes (UBC9), and E3 protein ligases. There are three types of SUMO proteins: SUMO1, SUMO2, and SUMO3 (Gareau and Lima, 2010; Qin et al., 2021). Tripartite motif-containing protein 28 (TRIM28) SUMOylates NLRP3 to inhibit K48 ubiquitination and the degradation of NLRP3, which promotes inflammasome activation (Qin et al., 2021). SUMO-specific proteases (SENP6 and SENP7) contribute to NLRP3 inflammasome activation, and MAPL SUMOylates NLRP3, leading to the suppression of inflammasome activation (Barry et al., 2018). SENP3 is a specific SUMO1 protease that deSUMOylates the NLRP3-Lys204 residue and leads to inactivation of NLRP3 (Shao L. et al., 2020).

### Alkylation

Several chemicals target NLRP3 ATPase and inhibit the activation of NLRP3 by alkylation, including parthenolide, BAY11-7085, BOT-4-one, 3,4-methylenedioxy-β-nitrostyrene (MNS), and acrylamide derivatives (Shim et al., 2017). Parthenolide directly alkylates Cys285 of caspase-1 p20 to inactive caspase-1 and the NLRP3 inflammasome, and BAY11-7085 inhibits NLRP3 (Juliana et al., 2010). BOT-4-one is an NLRP3-alkylating agent that significantly inhibits NLRP3 inflammasome activation by inhibiting the ATPase activity of NLRP3 and enhancing NLRP3 ubiquitination. However, the specific residues associated with alkylation are not clear and need further examination (Shim et al., 2017). MNS inhibits NLRP3 activation by alkylating NLRP3 and suppressing ATPase activity similarly to BAY11-7085 (He et al., 2014).

### S-nitrosylation

Inducible nitric oxide synthase (iNOS) causes the S-nitrosylation of NLRP3, which plays a negative role in NLRP3 inflammasome assembly and IL-1β maturation (Mishra et al., 2013). The S-nitrosylation of caspase-1 may contribute to the inhibitory effect of NO on absent in melanoma 2 (AIM2) and NLR family CARD domain-containing 4 protein (NLRC4; Hernandez-Cuellar et al., 2012). NO directly S-nitrosylates the inflammasome and downregulates NLRP3 activation and IL-1β release (Park et al., 2013).

## Treatments

NLRP3 inflammasome activation and the induction of canonical pyroptosis are related to PNDs. Any blockade of the pathway, such as upstream of the NLRP3 inflammasome, the formation of the NLRP3 inflammasome, and its downstream factors (GSDMD, IL-1β, and IL-18), inhibits pyroptosis and may reverse the progression of PNDs. We described some drugs, chemicals, new materials, and technologies to potentially treat the activation of NLRP3 in PNDs.

### Drugs

Except for the molecules or proteins introduced in the PTM section that target the NLRP3 inflammasome pathway, some drugs have therapeutic efficacy evidence to alleviate cognitive dysfunction and are presented in Table 1.

**Table 1:** NLRP3 inflammasome-associated drugs.

Drug	Functional mechanism or site	Function and effect
Suberoylanilide hydroxamic acid (SAHA)	Increased histone H3 and H4 acetylation Fang et al. (2021)	Autophagy↑ NLRP3 inflammasome↓ Sevoflurane-induced cognitive decline↓
Dexmedetomidine (DEX)	Autophagy–ubiquitin pathway of NLRP3 inflammasome Zhang et al. (2021b)	NLRP3↓, CASP1↓, and IL-1β↓ in the hippocampus Learning and memory ability impairment↓
	NF-κB pathway Wang L. et al. (2017)	mRNA levels of hippocampal IL-1β, IL-6, and TNF-α↓
	α2-AR/AMPK/mTOR pathway Yang et al. (2020)	Autophagy↑
	Reducing the oxidative stress response Cho et al. (2022)	NLRP3 inflammasome information↓ cognitive impairment in POCD mice↓
Atorvastatin, an HMG-CoA reductase inhibitor	Inhibiting the NF-κB-NLRP3 inflammasome pathway protecting the integrity of the blood–brain barrier (BBB) Liu P. et al. (2021)	IL-1β↓, IL-6↓, TNF-α↓ in the hippocampus and serum NLRP3 inflammasome↓ in the hippocampus NF-κB pathway↓
PHA 568487, a nAChR agonist	Inhibiting NF-κB activation Terrando et al. (2011)	NF-κB activation↓ in bone marrow-derived macrophage (BMDM) postoperative cognitive impairment↓
Elamipretide, as a mitochondrial-targeted peptide	Improving mitochondrial function Zuo et al. (2020)	NLRP3 inflammasome-induced pyroptosis ↓ impairment of synaptic and cognitive↓
Lidocaine	Reducing mitochondrial damage Li et al. (2019)	Cognitive impairment↓ Mitochondrial structure damage↓
ChIV	ROS Shao A. et al. (2020).	NLRP3-caspase-1 pathway↓ ROS production ↓
Ginsenosides (Rh1, Rg3) of Korean red ginseng (RGE)	ROS and intracellular calcium ions Kim et al. (2014).	Activation of NLRP3 and AIM2↓ IL-1β ↓ pyroptosis↓
Salidroside (Sal)	Inhibiting the TLR4/MyD88/NF-κB signaling pathways; TXNIP/NLRP3/caspase-1 pathways Zhang X. et al. (2020)	IL-1β, IL-18 and Gasdermin D↓ in PD mice TLR4, MyD88, p-IκBα, and p-NF-κB↓ in LPS-induced BV2 cell pyroptosis↓
	TLR4/NF-κB/NLRP3/caspase-1 signaling pathway Cai et al. (2021)	TLR4, MyD88, NF-κB, P-NF-κB, NLRP3, ASC, cleaved Caspase-1, cleaved GSDMD, IL-1β, and IL-18↓ *in vitro* pyroptosis↓
Prussian blue nanozyme (PBzyme)	Scavenging ROS Ma X. et al. (2022)	IL-1β, IL-6, and TNF-α, NLRP3, cleaved caspase-1, GSDMD, cleaved GSDMD, and ROS generation↓ in PD mice.
MCC950	Inhibiting NLRP3 Fan et al. (2018), Coll et al. (2015)	NLRP3-induced pyroptosis↓
MM01	ASC-CARD domain-related residuals, such as Trp-169. Soriano-Teruel et al. (2021).	ASC-mediated inflammatory signaling(NLRP1/NLRC4)↓ ASC oligomerization and pro-caspase activation↓
U50488H, k-opioid receptor agonist	The NLRP3/caspase-1 pathway Song et al. (2021)	NLRP3 and associated proteins↓ Pyroptosis↓ cerebral and cognitive impairment↓
Annexin-A1 (ANXA1) tripeptide	NLRP3 inflammasome Zhang et al. (2022)	PNDs-like behavior ↓ ASC, NLRP3, and IL-1β↓
Necrosulfonamide (NSA)	Cys191 on GSDMD Rathkey et al. (2018).	Oligomerization of GSDMD dimer↓; SDMD pore formation↓ in murine and human cells; pyroptotic cell death↓;does not interfere with inflammasome formation.
	Cleavage of caspase-1 Hu et al. (2020).	Processing of caspase-1, IL-1β, and GSDMD↓ in cells
Disulfiram (DSF)	GSDMD pore formation Hu et al. (2020).	No apparent effect on ASC speck formation, the cleavage of caspase-1, GSDMD, and pro-IL-1β IL-1β, TNF, and IL-6↓in the serum of mice
Dimethyl fumarate (DMF)	Succination of GSDMD Humphries et al. (2020).	IL-1β↓ *in vivo*; GSDMD-caspases interaction, processing, and the oligomerization of GSDMD↓
Anakinra and canakinumab	IL-1β antagonist Dinarello et al. (2012)	

#### Inhibition of factors upstream of The NLRP3 inflammasome

Suberoylanilide hydroxamic acid (SAHA), a histone deacetylase inhibitor, suppresses NLRP3 inflammasome activation by enhancing autophagy and ameliorating sevoflurane-induced PNDs (Fang et al., 2021). Dexmedetomidine (DEX) is a commonly used analgesic in the clinic, that reduces the occurrence of PNDs. DEX blocks NLRP3 inflammasome activation by inhibiting NF-κB, reducing the oxidative stress response, promoting NLRP3 inflammasome degradation, and inhibiting NLRP3 inflammasome activation *via* autophagy (Yang et al., 2020; Zhang et al., 2021b; Cho et al., 2022; Li et al., 2022).

Atorvastatin (an HMG-CoA reductase inhibitor) (Liu P. et al., 2021) and PHA 568487 [a nicotinic acetylcholine receptor (nAChR) agonist] (Terrando et al., 2011) prevent PNDs by inhibiting NF-κB activation and mitochondrial dysfunction. Elamipretide attenuates NLRP3 inflammasome-induced pyroptosis and impairs synaptic and cognitive function by improving mitochondrial function (Zuo et al., 2020). Lidocaine also improves the cognitive injury caused by isoflurane by reducing mitochondrial dysfunction (Li et al., 2019). Several traditional medicines have clinical effects, such as Chikusetsu saponin IVa (ChIV) and ginsenosides (Rh1, Rg3) from Korean red ginseng (RGE), which downregulate the NLRP3 pathway by reducing the production of ROS (Kim et al., 2014; Shao A. et al., 2020). Salidroside (Sal) and Prussian blue nanozyme (PBzyme) effectively supreess the activation of the NLRP3 inflammasome and pyroptosis *via* their ROS-scavenging properties in mouse models of Parkinson’s disease and AD (Zhang X. et al., 2020; Cai et al., 2021; Ma X. et al., 2022), and these agents may be used as treatments in a PNDs model.

#### Inhibition of NLRP3 inflammasome activation

Inhibiting the components of the NLRP3 inflammasome, such as ASC, NLRP3 protein, and pro-caspase-1, effectively suppress its activation. Because ASC and caspase also play roles in other inflammatory processes, NLRP3 is specific to the canonical pathway and a better target. MCC950 is a specific NLRP3 inhibitor that effectively inhibits NLRP3-associated downstream events to prevent PNDs in mouse models (Coll et al., 2015; Fan et al., 2018). MM01 inhibited inflammation by preventing ASC oligomerization and pro-caspase activation in mouse peritonitis models, but its pharmacological action and safety need further confirmation (Soriano-Teruel et al., 2021). U50488H is a κ-opioid receptor agonist and annexin-A1 (ANXA1) tripeptide that effectively inhibits pyroptosis and improves PNDs by targeting the NLRP3 inflammasome *via* an unclear mechanism (Song et al., 2021; Zhang et al., 2022).

#### Inhibition of factors downstream of The NLRP3 inflammasome

GSDMD is a characteristic sign and key protein of pyroptosis, and it is a common substrate of the canonical and noncanonical NLRP3 inflammasome activation pathways. GSDMD is a specific and advantageous target, and any treatment focusing on GSDMD may alleviate pyroptosis and prevent PNDs. Dimethyl fumarate (DMF), disulfiram (DSF), and necrosulfonamide (NSA) inhibit GSDMD by modifying Cys191 (Cys192 in mice) residues (Liu X. et al., 2021). NSA inhibits GSDMD pore formation by hindering the oligomerization of GSDMD dimers but does not affect the cleavage of GSDMD (Rathkey et al., 2018). However, Liu et al. demonstrated that the inhibitory effects of NSA and BAY11-7082 involved inhibition of the cleavage of caspase-1, IL-1β, and GSDMD. DSF is the only direct inhibitor of GSDMD, and it prevented pyroptosis by inhibiting GSDMD pore formation. However, DSF did not inhibit GSDMD cleavage or caspase-11. In contrast, NSA, Bay11-7,082, DMF, and z-VAD-fmk have little or no effect on GSDMD inhibition (Hu et al., 2020). DMF plays a role in inhibiting pyroptosis by succinate GSDMD, which prevents the interaction of GSDMD and caspase (Humphries et al., 2020). Rats treated with DSF or NFA exhibited attenuated cognitive impairment caused by sevoflurane neurotoxicity (Wen-Yuan et al., 2022).

Inhibition of cytokines also prevents PNDs. The IL-1β antagonists anakinra and canakinumab are less effective than the inhibitors of GSDMD because these agents only inhibit a single cytokine (Liu X. et al., 2021).

### Technologies

#### Gene and gene editing technology

Genes affect protein expression and influence the mechanism of PNDs. Knockdown of *SETD7* effectively suppressed NLRP3-dependent pyroptosis and reversed isoflurane-induced cognitive dysfunction (Ma C. et al., 2022). Overexpression of *DUSP14* partially suppressed the NLRP3-caspase-1 pathway by reducing the levels of cytokines and pyroptosis to ultimately ameliorate cognitive dysfunction (Que et al., 2020). *HTR2A* upregulation reduced the expression of pyroptosis-related genes (cleaved GSDMD, NLRP3, and ASC) and suppressed pyroptosis in hippocampal neurons in PNDs rats (Wu et al., 2022).

Clustered regularly interspaced short palindromic repeats/CRISPR-associated nuclease9 (CRISPR/Cas9) is a new gene editing technology that targets any genomic locus using only a complex nuclease protein with short RNA as a site-specific endonuclease. CRISPR/Cas9 has been used in the field of cancer research to edit genomes to examine the mechanisms of tumorigenesis and development (Zhang H. et al., 2021) and explore the complex interactions and disruptions in genes that contribute to Huntington’s disease (HD, a neurodegenerative disease; Tabrizi et al., 2020) and AD (Schrauben et al., 2020). AD and PNDs may have similar mechanisms and gene effects, and CRISPR/Cas9 may be used in future studies of PNDs.

#### Targeted protein degradation (TPD)

TPD technology uses two naturally occurring protein degradation systems in cells (the ubiquitination proteasome system and lysosomal degradation pathway) to achieve specific and efficient degradation of disease-related proteins and facilitate disease treatment. Proteolysis targeting chimera (PROTAC) technology is based on the E3 ligase of the ubiquitination proteasome system, and it induces the degradation of a given protein of interest (Nalawansha and Crews, 2020). Compared to traditional small-molecule inhibitors, drugs based on TPD technology are less restricted in the selection of target proteins and act on “nondrug-resistant” proteins. Compared to gene or mRNA translation, TPD drugs are specific, fast, and free from posttranslational modifications. TPD experienced explosive growth in cancer research and entered clinical development as a cancer therapy (Dale and Cheng, 2021).

As misfolded protein aggregates are associated with many neurodegenerative diseases, TPD was applied to target proteins of interest to treat these diseases, such as AD, HD, and Parkinson’s disease (Hyun and Shin, 2021; Benn et al., 2022). Tau proteins cause Aβ aggregation and play a role in AD, which is also the potential role of PNDs (Yu et al., 2020). In 2016, Chu et al. reported the TPD for tau protein using a peptide-based PROTAC compound and reduced the neurotoxicity of Aβ through TU006-mediated lowering of the tau protein in an AD transgenic mouse model (Chu et al., 2016). M. C. Silva et al. synthesized the degrader compound QC-01-175, which binds CRBN E3 ligase and tau protein to induce ubiquitination of tau protein and proteasomal degradation in frontotemporal dementia neuronal cell models (Silva and Ferguson, 2019). Small-molecule PROTACs have been shown to induce the degradation of huntingtin in fibroblasts from HD patients by E3 ligase (Yamashita et al., 2020).

As PROTAC technology is based on the E3 ligase of ubiquitination, which also moderates NLRP3 inflammasome activation (as introduced in the previous PTMs section), it may be used in future studies of PNDs focusing on NLRP3-related proteins. At least now, there is no report of TPD safety and application in PNDs studies. All of these findings need to be confirmed by future research.

#### Nanotechnology

Nanotechnology is a rapidly emerging field that manipulates assorted synthetic and naturally occurring materials in nanoscale dimensions (1–1,000 nm), and it is used in tissue regeneration, drug delivery, and pharmaceuticals (Sridhar et al., 2015). It uses a variety of materials to synthesize functional organizations, such as polymers, lipids, viruses, and organometallic compounds. Nanoparticles (NPs) link biological molecules or ligands that act as address tags to direct the NPs to specific sites and specific cellular organelles or specifically follow the movement of individual proteins or RNA molecules (Gupta et al., 2019). NPs have been widely used in research on cancer to carry different medicines that induce pyroptosis for cancer immunotherapy (Ding et al., 2021) and inhibit pyroptosis in sepsis (Liu B. et al., 2021; Chen et al., 2022). Yao et al. reported that NPs carrying hesperidin, which is an extract in citrus fruits, effectively identified inflammatory neutrophils and quickly accumulated in the injured area to reduce the secretion of inflammatory factors in traumatic brain injury (Yao et al., 2022). NSA could bind directly to Cys191 of human GSDMD or to Cys192 of mouse GSDMD and then inhibit GSDMD pore formation and reduce the release of IL-1β, but had potential toxic organic solvents. Several types of porous NPs, mesoporous silica (MSN), porous cross-linked cyclodextrin carriers (CD-NP), and a mesoporous magnesium-phosphate carrier (MPC-NP), targeted delivered NSA to phagocytic cells and effectively inhibited GSDMD activation to regulate inflammatory responses (Boersma et al., 2022).

Various NPs or nanomedicines (NMs) have been synthesized in recent decades to exploit the existing physiological mechanisms of passage through the BBB, including receptor- and adsorptive-mediated transcytosis, which facilitate the transcellular transport of NPs from the blood to the brain, to explore their potential application in the diagnosis and therapy of AD (Gupta et al., 2019). Nanotechnology graphene oxide (GO)/graphene is a novel nanocarbon material that alleviates Aβ burden and improves learning and memory in a mouse model of AD (Xiao et al., 2016) and PNDs in mice (Zhang J. et al., 2020).

Although there is less research on the use of nanotechnology in PNDs treatment, nanotechnology will likely be used for PNDs treatment in the future based on emerging research of this new technology.

## Discussion

PNDs are frequent complications with cognitive impairment that is identified during the perioperative period and may be induced by surgery or anesthetic. PNDs occur more commonly in elderly patients. With the aging of the population and the increased demand for surgery, the foreseeable increase in PNDs will lead to increased morbidity, mortality, and a heavier burden on families and society (Androsova et al., 2015; Wei et al., 2019). The underlying mechanisms are complex and unclear, but inflammation may be the key mechanism of PNDs (Cibelli et al., 2010). Pyroptosis is an inflammatory form of programmed cell death that contributes to neuronal death, is associated with neurodegenerative diseases, such as AD and Parkinson’s disease, and is related to PNDs. NLRP3 inflammasome-induced canonical pyroptosis may be associated with the mechanism of PNDs (Zhang et al., 2021b). The present study primarily demonstrated NLRP3 inflammasome-related neuroinflammation in PNDs and the components and mechanisms of NLPR3 induced pyroptosis. The review introduced the PTMs of NLRP3 and suggested prevention strategies (drugs and new technology) by targeting NLRP3 inflammasomes in future studies of PNDs.

The NLRP3 inflammasome is a multiprotein complex that includes NLRP3 protein, ASC, and pro-caspase-1 (Swanson et al., 2019). There are three kinds of NLRP3 inflammasome activation pathways, as presented in Figure 1. There are several targets that interfere with NLRP3-dependent pyroptosis, including upstream of the NLRP3 inflammasome, the inflammasome itself, and downstream of the inflammasome. NF-κB is a critical upstream target of the NLRP3 inflammasome (Bauernfeind et al., 2009). Activation of the NF-κB pathway upregulates the transcription of NLRP3 and IL-1β, which are necessary for canonical NLRP3 pathway activation (Bauernfeind et al., 2009). Mitochondrial dysfunction (Zhou et al., 2011; Zuo et al., 2020), ionic flux (Muñoz-Planillo et al., 2013; Swanson et al., 2019), trans-Golgi disassembly (Gaidt et al., 2016), and plasma membrane rupture (Schorn et al., 2011) are also likely upstream events of the NLRP3 inflammasome. Inhibition of these pathways also effectively downregulates the expression of the NLRP3 inflammasome. However, using these pathways as treatment targets may result in a lack of specificity because they also participate in other signaling pathways. Targeting the assembly of the inflammasome is a highly specific strategy, but there are few definite drugs at present. Notably, GSDMD is an indispensable target of pyroptosis and may be a specific target to control pyroptosis.

PTMs are an emerging and potential research field because they modify multiple proteins. PTMs occur at many steps in pyroptosis pathway proteins, such as NLRP3 and gasdermins (Fischer et al., 2021). We used the NLRP3 inflammasome as an example to briefly introduce PTMs, including ubiquitination, phosphorylation, SUMOylation alkylation, and S-nitrosylation. Some molecules that influence the PTMs of NLRP3 are presented in Figure 2. Different PTMs work interactively, and inactivating NLRP3 may be a potential treatment strategy for PNDs. The specific functional site and mechanisms that may be the target of PNDs need further research.

Based on current knowledge of NLPR3-induced pyroptosis, we briefly listed some drugs that decrease pyroptosis and release PNDs, focusing on upstream of the NLRP3 inflammasome, the inflammasome itself, and downstream of the inflammasome (Table 1). Some new technologies are emerging, including gene editing, TPD, and nanotechnology. TPD performs in a highly specific and advanced manner using the PTM mechanism to regulate the degradation of particular proteins. Nanotechnology is a rapidly emerging program that is applied in tissue regeneration, drug delivery, and pharmaceuticals (Sridhar et al., 2015). Various NPs or NMs have been synthesized to exploit the existing physiological mechanisms of disease treatments by carrying the drug through the BBB to provide a high concentration at the target receptor and prolonging the drug effect time. Although there is less research on the application of new technologies in PNDs, these technologies are widely used in the research of cancer and AD (Xiao et al., 2016; Dale and Cheng, 2021; Ding et al., 2021). Therefore, their use for PNDs treatment is highly likely in the near future.

In conclusion, NLRP3 inflammasomes induce the canonical pyroptosis pathway, which contributes to PNDs and is an effective therapeutic target for the treatment of PNDs. Based on the mechanisms of the NLRP3 pathway, any activation of NLRP3 upstream promoting factors, the NLRP3 inflammasome, and its downstream factors (GSDMD, IL-1β, and IL-18) may be treatment targets for PNDs. The current review systematically illustrated some drugs, chemicals, new materials, and technologies that inhibit the activation of NLRP3 and may be used to treat PNDs patients in the future.

## Author contributions

FB conceived the original idea. JL wrote the manuscript with support from LL, JH, and JX. All authors contributed to the article and approved the submitted version.

## Funding

The study was supported by Experimental Animal of Public Welfare Research Project of Zhejiang Province (grand number LGD20H090006, FB) and The Medical Scientific Research Foundation of Zhejiang Province (grand number 2020KY626, FB), China.

## Conflict of interest

The authors declare that the research was conducted in the absence of any commercial or financial relationships that could be construed as a potential conflict of interest.

## Publisher’s note

All claims expressed in this article are solely those of the authors and do not necessarily represent those of their affiliated organizations, or those of the publisher, the editors and the reviewers. Any product that may be evaluated in this article, or claim that may be made by its manufacturer, is not guaranteed or endorsed by the publisher.

## References

[ref1] Akther M. Haque M. E. Park J. Kang T. B. Lee K. H. (2021). NLRP3 Ubiquitination-A new approach to target NLRP3 Inflammasome activation. Int. J. Mol. Sci. 22:8780. doi: 10.3390/ijms22168780, PMID: 34445484 PMC8395773

[ref2] Androsova G. Krause R. Winterer G. Schneider R. (2015). Biomarkers of postoperative delirium and cognitive dysfunction. Front. Aging Neurosci. 7:112. doi: 10.3389/fnagi.2015.00112, PMID: 26106326 PMC4460425

[ref3] Barry R. John S. W. Liccardi G. Tenev T. Jaco I. Chen C. H.. (2018). Sumo-mediated regulation of NLRP3 modulates inflammasome activity. Nat. Commun. 9:3001. doi: 10.1038/s41467-018-05321-2 30069026 PMC6070540

[ref4] Bauernfeind F. G. Horvath G. Stutz A. Alnemri E. S. Macdonald K. Speert D.. (2009). Cutting edge: Nf-kappaB activating pattern recognition and cytokine receptors license NLRP3 inflammasome activation by regulating NLRP3 expression. J. Immunol. 183, 787–791. doi: 10.4049/jimmunol.0901363, PMID: 19570822 PMC2824855

[ref5] Beckwith K. S. Beckwith M. S. Ullmann S. Sætra R. S. Kim H.. (2020). Plasma membrane damage causes NLRP3 activation and pyroptosis during mycobacterium tuberculosis infection. Nat. Commun. 11:2270. doi: 10.1038/s41467-020-16143-6, PMID: 32385301 PMC7210277

[ref6] Benn J. A. Mukadam A. S. Mcewan W. A. (2022). Targeted protein degradation using intracellular antibodies and its application to neurodegenerative disease. Semin. Cell Dev. Biol. 126, 138–149. doi: 10.1016/j.semcdb.2021.09.012, PMID: 34654628

[ref7] Boersma B. Möller K. Wehl L. Puddinu V. Huard A. Fauteux-Daniel S.. (2022). Inhibition of Il-1β release from macrophages targeted with necrosulfonamide-loaded porous nanoparticles. J. Control. Release 351, 989–1002. doi: 10.1016/j.jconrel.2022.09.063, PMID: 36202154

[ref8] Cai Y. Chai Y. Fu Y. Wang Y. Zhang Y. Zhang X.. (2021). Salidroside ameliorates Alzheimer's disease by targeting NLRP3 inflammasome-mediated pyroptosis. Front. Aging Neurosci. 13:809433. doi: 10.3389/fnagi.2021.809433, PMID: 35126093 PMC8814655

[ref9] Chen Y. Luo R. Li J. Wang S. Ding J. Zhao K.. (2022). Intrinsic radical species scavenging activities of tea polyphenols nanoparticles block pyroptosis in endotoxin-induced sepsis. ACS Nano 16, 2429–2441. doi: 10.1021/acsnano.1c08913, PMID: 35133795

[ref10] Chi H. Kawano T. Tamura T. Iwata H. Takahashi Y. Eguchi S.. (2013). Postoperative pain impairs subsequent performance on a spatial memory task via effects on N-methyl-D-aspartate receptor in aged rats. Life Sci. 93, 986–993. doi: 10.1016/j.lfs.2013.10.028, PMID: 24211778

[ref11] Cho I. Koo B. N. Kim S. Y. Park S. Kim E. J. Kam E. H.. (2022). Neuroprotective effect of dexmedetomidine against postoperative cognitive decline via NLRP3 inflammasome signaling pathway. Int. J. Mol. Sci. 23:8806. doi: 10.3390/ijms23158806, PMID: 35955939 PMC9369249

[ref12] Chu T. T. Gao N. Li Q. Q. Chen P. G. Yang X. F. Chen Y. X.. (2016). Specific knockdown of endogenous tau protein by peptide-directed ubiquitin-proteasome degradation. Cell Chem. Biol. 23, 453–461. doi: 10.1016/j.chembiol.2016.02.016, PMID: 27105281

[ref13] Cibelli M. Fidalgo A. R. Terrando N. Ma D. Monaco C. Feldmann M.. (2010). Role of interleukin-1beta in postoperative cognitive dysfunction. Ann. Neurol. 68, 360–368. doi: 10.1002/ana.22082, PMID: 20818791 PMC4836445

[ref14] Coll R. C. Robertson A. A. Chae J. J. Higgins S. C. Muñoz-Planillo R. Inserra M. C.. (2015). A small-molecule inhibitor of the NLRP3 inflammasome for the treatment of inflammatory diseases. Nat. Med. 21, 248–255. doi: 10.1038/nm.3806, PMID: 25686105 PMC4392179

[ref15] Cowie A. M. Menzel A. D. Ohara C. Lawlor M. W. Stucky C. L. (2019). Nod-like receptor protein 3 inflammasome drives postoperative mechanical pain in a sex-dependent manner. Pain 160, 1794–1816. doi: 10.1097/j.pain.0000000000001555, PMID: 31335648 PMC6662742

[ref16] Dale B. Cheng M. (2021). Advancing targeted protein degradation for cancer therapy. Nat. Rev. Cancer 21, 638–654. doi: 10.1038/s41568-021-00365-x, PMID: 34131295 PMC8463487

[ref17] Dinarello C. A. Simon A. Van Der Meer J. W. (2012). Treating inflammation by blocking interleukin-1 in a broad spectrum of diseases. Nat. Rev. Drug Discov. 11, 633–652. doi: 10.1038/nrd3800, PMID: 22850787 PMC3644509

[ref18] Ding B. Sheng J. Zheng P. Li C. Li D. Cheng Z.. (2021). Biodegradable upconversion nanoparticles induce pyroptosis for cancer immunotherapy 21, 8281–8289. doi: 10.1021/acs.nanolett.1c02790, PMID: 34591494

[ref19] Evered L. Silbert B. Knopman D. S. Scott D. A. Dekosky S. T. Rasmussen L. S.. (2018). Recommendations for the nomenclature of cognitive change associated with anaesthesia and surgery-20181. J. Alzheimers Dis. 66, 1–10. doi: 10.3233/jad-189004, PMID: 30347621

[ref20] Evered L. Silbert B. Scott D. A. Ames D. Maruff P. Blennow K. (2016). Cerebrospinal fluid biomarker for Alzheimer disease predicts postoperative cognitive dysfunction. Anesthesiology 124, 353–361. doi: 10.1097/aln.0000000000000953, PMID: 26580833

[ref21] Fan Y. Du L. Fu Q. Zhou Z. Zhang J. Li G.. (2018). Inhibiting the NLRP3 inflammasome with Mcc950 ameliorates Isoflurane-induced pyroptosis and cognitive impairment in aged mice. Front. Cell. Neurosci. 12:426. doi: 10.3389/fncel.2018.00426, PMID: 30524241 PMC6262296

[ref22] Fan K. Yang J. Gong W. Y. Pan Y. C. Zheng P. Yue X. F. (2021). NLRP3 inflammasome activation mediates sleep deprivation-induced pyroptosis in mice. PeerJ 9:e11609. doi: 10.7717/peerj.11609, PMID: 34268006 PMC8269641

[ref23] Fang P. Chen C. Zheng F. Jia J. Chen T. Zhu J.. (2021). NLRP3 inflammasome inhibition by histone acetylation ameliorates sevoflurane-induced cognitive impairment in aged mice by activating the autophagy pathway. Brain Res. Bull. 172, 79–88. doi: 10.1016/j.brainresbull.2021.04.016, PMID: 33895270

[ref24] Fischer F. A. Chen K. W. Bezbradica J. S. (2021). Posttranslational and therapeutic control of gasdermin-mediated pyroptosis and inflammation. Front. Immunol. 12:661162. doi: 10.3389/fimmu.2021.661162, PMID: 33868312 PMC8050342

[ref25] Fu Q. Li J. Qiu L. Ruan J. Mao M. Li S.. (2020). Inhibiting NLRP3 inflammasome with MCC950 ameliorates perioperative neurocognitive disorders, suppressing neuroinflammation in the hippocampus in aged mice. Int. Immunopharmacol. 82:106317. doi: 10.1016/j.intimp.2020.106317, PMID: 32087497

[ref26] Gaidt M. M. Ebert T. S. Chauhan D. Schmidt T. Schmid-Burgk J. L. Rapino F.. (2016). Human monocytes engage an alternative inflammasome pathway. Immunity 44, 833–846. doi: 10.1016/j.immuni.2016.01.012, PMID: 27037191

[ref27] Gareau J. R. Lima C. D. (2010). The SUMO pathway: emerging mechanisms that shape specificity, conjugation and recognition. Nat. Rev. Mol. Cell Biol. 11, 861–871. doi: 10.1038/nrm3011 21102611 PMC3079294

[ref28] Glickman M. H. Ciechanover A. (2002). The ubiquitin-proteasome proteolytic pathway: destruction for the sake of construction. Physiol. Rev. 82, 373–428. doi: 10.1152/physrev.00027.2001, PMID: 11917093

[ref29] Gong T. Jiang W. Zhou R. (2018). Control of inflammasome activation by phosphorylation. Trends Biochem. Sci. 43, 685–699. doi: 10.1016/j.tibs.2018.06.008 30049633

[ref30] Guo C. Xie S. Chi Z. Zhang J. Liu Y. Zhang L.. (2016). Bile acids control inflammation and metabolic disorder through inhibition of NLRP3 inflammasome. Immunity 45, 802–816. doi: 10.1016/j.immuni.2016.09.008 27692610

[ref31] Gupta J. Fatima M. T. Islam Z. Khan R. H. Uversky V. N. Salahuddin P. (2019). Nanoparticle formulations in the diagnosis and therapy of Alzheimer's disease. Int. J. Biol. Macromol. 130, 515–526. doi: 10.1016/j.ijbiomac.2019.02.156, PMID: 30826404

[ref32] Han X. Sun S. Sun Y. Song Q. Zhu J. Song N.. (2019). Small molecule-driven NLRP3 inflammation inhibition via interplay between ubiquitination and autophagy: implications for Parkinson disease. Autophagy 15, 1860–1881. doi: 10.1080/15548627.2019.1596481, PMID: 30966861 PMC6844502

[ref33] He Y. Hara H. Núñez G. (2016). Mechanism and regulation of NLRP3 inflammasome activation. Trends Biochem. Sci. 41, 1012–1021. doi: 10.1016/j.tibs.2016.09.002, PMID: 27669650 PMC5123939

[ref34] He X. F. Li L. L. Xian W. B. Li M. Y. Zhang L. Y. Xu J. H.. (2021). Chronic colitis exacerbates NLRP3-dependent neuroinflammation and cognitive impairment in middle-aged brain. J. Neuroinflammat. 18:153. doi: 10.1186/s12974-021-02199-8, PMID: 34229722 PMC8262017

[ref35] He Y. Varadarajan S. Muñoz-Planillo R. Burberry A. Nakamura Y. Núñez G. (2014). 3,4-methylenedioxy-β-nitrostyrene inhibits NLRP3 inflammasome activation by blocking assembly of the inflammasome. J. Biol. Chem. 289, 1142–1150. doi: 10.1074/jbc.M113.515080, PMID: 24265316 PMC3887181

[ref36] He H. J. Wang Y. Le Y. Duan K. M. Yan X. B. Liao Q.. (2012). Surgery upregulates high mobility group box-1 and disrupts the blood-brain barrier causing cognitive dysfunction in aged rats. CNS Neurosci. Ther. 18, 994–1002. doi: 10.1111/cns.12018, PMID: 23078219 PMC6493557

[ref37] Hernandez-Cuellar E. Tsuchiya K. Hara H. Fang R. Sakai S. Kawamura I.. (2012). Cutting edge: nitric oxide inhibits the NLRP3 inflammasome. J. Immunol. 189, 5113–5117. doi: 10.4049/jimmunol.1202479, PMID: 23100513

[ref38] Hou J. Zhao R. Xia W. Chang C. W. You Y. Hsu J. M.. (2020). Pd-L1-mediated gasdermin C expression switches apoptosis to pyroptosis in cancer cells and facilitates tumour necrosis. Nat. Cell Biol. 22, 1264–1275. doi: 10.1038/s41556-020-0575-z, PMID: 32929201 PMC7653546

[ref39] Hu J. J. Liu X. Xia S. Zhang Z. Zhang Y. Zhao J.. (2020). Fda-approved disulfiram inhibits pyroptosis by blocking gasdermin D pore formation. Nat. Immunol. 21, 736–745. doi: 10.1038/s41590-020-0669-6, PMID: 32367036 PMC7316630

[ref40] Huang Y. Xu W. Zhou R. (2021). NLRP3 inflammasome activation and cell death. Cell. Mol. Immunol. 18, 2114–2127. doi: 10.1038/s41423-021-00740-6, PMID: 34321623 PMC8429580

[ref41] Humphries F. Shmuel-Galia L. Ketelut-Carneiro N. Li S. Wang B. Nemmara V. V.. (2020). Succination inactivates gasdermin D and blocks pyroptosis. Science 369, 1633–1637. doi: 10.1126/science.abb9818, PMID: 32820063 PMC8744141

[ref42] Hyun S. Shin D. (2021). Chemical-mediated targeted protein degradation in neurodegenerative diseases. Life 11:607. doi: 10.3390/life11070607, PMID: 34202541 PMC8305580

[ref43] Ising C. Venegas C. Zhang S. Scheiblich H. Schmidt S. V. Vieira-Saecker A.. (2019). NLRP3 inflammasome activation drives tau pathology. Nature 575, 669–673. doi: 10.1038/s41586-019-1769-z, PMID: 31748742 PMC7324015

[ref44] Ji M. H. Yuan H. M. Zhang G. F. Li X. M. Dong L. Li W. Y.. (2013). Changes in plasma and cerebrospinal fluid biomarkers in aged patients with early postoperative cognitive dysfunction following total hip-replacement surgery. J. Anesth. 27, 236–242. doi: 10.1007/s00540-012-1506-3, PMID: 23085747

[ref45] Juliana C. Fernandes-Alnemri T. Wu J. Datta P. Solorzano L. Yu J. W.. (2010). Anti-inflammatory compounds parthenolide and bay 11-7082 are direct inhibitors of the inflammasome. J. Biol. Chem. 285, 9792–9802. doi: 10.1074/jbc.M109.082305, PMID: 20093358 PMC2843228

[ref46] Kalisvaart K. J. Vreeswijk R. De Jonghe J. F. Van Der Ploeg T. Van Gool W. A. Eikelenboom P. (2006). Risk factors and prediction of postoperative delirium in elderly hip-surgery patients: implementation and validation of a medical risk factor model. J. Am. Geriatr. Soc. 54, 817–822. doi: 10.1111/j.1532-5415.2006.00704.x, PMID: 16696749

[ref47] Kim J. Ahn H. Han B. C. Lee S. H. Cho Y. W. Kim C. H.. (2014). Korean red ginseng extracts inhibit NLRP3 and Aim2 inflammasome activation. Immunol. Lett. 158, 143–150. doi: 10.1016/j.imlet.2013.12.017, PMID: 24418475

[ref48] Kotekar N. Shenkar A. Nagaraj R. (2018). Postoperative cognitive dysfunction – current preventive strategies. Clin. Interv. Aging 13, 2267–2273. doi: 10.2147/cia.S133896, PMID: 30519008 PMC6233864

[ref49] Lawlor K. E. Vince J. E. (2014). Ambiguities in NLRP3 inflammasome regulation: is there a role for mitochondria? Biochim. Biophys. Acta 1840, 1433–1440. doi: 10.1016/j.bbagen.2013.08.014, PMID: 23994495

[ref50] Li Z. Zhu Y. Kang Y. Qin S. Chai J. (2022). Neuroinflammation as the underlying mechanism of postoperative cognitive dysfunction and therapeutic strategies. Front. Cell. Neurosci. 16:843069. doi: 10.3389/fncel.2022.843069, PMID: 35418837 PMC8995749

[ref51] Li J. Zhu X. Yang S. Xu H. Guo M. Yao Y.. (2019). Lidocaine attenuates cognitive impairment after Isoflurane anesthesia by reducing mitochondrial damage. Neurochem. Res. 44, 1703–1714. doi: 10.1007/s11064-019-02799-0, PMID: 30989480

[ref52] Lian X. Zhu Q. Sun L. Cheng Y. (2021). Effect of anesthesia/surgery on gut microbiota and fecal metabolites and their relationship with cognitive dysfunction. Front. Syst. Neurosci. 15:655695. doi: 10.3389/fnsys.2021.655695, PMID: 34483850 PMC8416053

[ref53] Liu P. Gao Q. Guan L. Hu Y. Jiang J. Gao T.. (2021). Atorvastatin attenuates surgery-induced Bbb disruption and cognitive impairment partly by suppressing Nf-κB pathway and NLRP3 inflammasome activation in aged mice. Acta Biochim. Biophys. Sin. Shanghai 53, 528–537. doi: 10.1093/abbs/gmab022, PMID: 33674828

[ref54] Liu B. Li X. Yu H. Shi X. Zhou Y. Alvarez S.. (2021). Therapeutic potential of garlic chive-derived vesicle-like nanoparticles in NLRP3 inflammasome-mediated inflammatory diseases. Theranostics 11, 9311–9330. doi: 10.7150/thno.60265, PMID: 34646372 PMC8490522

[ref55] Liu X. Xia S. Zhang Z. Wu H. Lieberman J. (2021). Channelling inflammation: gasdermins in physiology and disease. Nat. Rev. Drug Discov. 20, 384–405. doi: 10.1038/s41573-021-00154-z 33692549 PMC7944254

[ref56] Liu X. Yu Y. Zhu S. (2018). Inflammatory markers in postoperative delirium (pod) and cognitive dysfunction (Pocd): a meta-analysis of observational studies. PLoS One 13:e0195659. doi: 10.1371/journal.pone.0195659, PMID: 29641605 PMC5895053

[ref57] Liu X. Zhang Z. Ruan J. Pan Y. Magupalli V. G. Wu H.. (2016). Inflammasome-activated gasdermin D causes pyroptosis by forming membrane pores. Nature 535, 153–158. doi: 10.1038/nature18629, PMID: 27383986 PMC5539988

[ref58] Lopez-Castejon G. (2020). Control of the inflammasome by the ubiquitin system. FEBS J. 287, 11–26. doi: 10.1111/febs.15118, PMID: 31679183 PMC7138099

[ref59] Lotze M. T. Tracey K. J. (2005). High-mobility group box 1 protein (Hmgb1): nuclear weapon in the immune arsenal. Nat. Rev. Immunol. 5, 331–342. doi: 10.1038/nri1594, PMID: 15803152

[ref60] Ma X. Hao J. Wu J. Li Y. Cai X. Zheng Y. (2022). Prussian blue nanozyme as a pyroptosis inhibitor alleviates neurodegeneration. Adv. Mater. 34:e2106723. doi: 10.1002/adma.202106723, PMID: 35143076

[ref61] Ma C. Yu X. Li D. Fan Y. Tang Y. Tao Q.. (2022). Inhibition of set domain-containing (lysine methyltransferase) 7 alleviates cognitive impairment through suppressing the activation of nod-like receptor protein 3 inflammasome in isoflurane-induced aged mice. Hum. Exp. Toxicol. 41:9603271211061497. doi: 10.1177/09603271211061497, PMID: 35187972

[ref62] Mangan M. S. J. Olhava E. J. Roush W. R. Seidel H. M. Glick G. D. Latz E. (2018). Targeting the NLRP3 inflammasome in inflammatory diseases. Nat. Rev. Drug Discov. 17, 588–606. doi: 10.1038/nrd.2018.97 30026524

[ref63] Mishra B. B. Rathinam V. A. Martens G. W. Martinot A. J. Kornfeld H. Fitzgerald K. A.. (2013). Nitric oxide controls the immunopathology of tuberculosis by inhibiting NLRP3 inflammasome-dependent processing of Il-1β. Nat. Immunol. 14, 52–60. doi: 10.1038/ni.2474, PMID: 23160153 PMC3721324

[ref64] Mortimer L. Moreau F. Macdonald J. A. Chadee K. (2016). NLRP3 inflammasome inhibition is disrupted in a group of auto-inflammatory disease caps mutations. Nat. Immunol. 17, 1176–1186. doi: 10.1038/ni.3538, PMID: 27548431

[ref65] Moujalled D. Strasser A. Liddell J. R. (2021). Molecular mechanisms of cell death in neurological diseases. Cell Death Differ. 28, 2029–2044. doi: 10.1038/s41418-021-00814-y, PMID: 34099897 PMC8257776

[ref66] Muñoz-Planillo R. Kuffa P. Martínez-Colón G. Smith B. L. Rajendiran T. M. Núñez G. (2013). K^+^ efflux is the common trigger of NLRP3 inflammasome activation by bacterial toxins and particulate matter. Immunity 38, 1142–1153. doi: 10.1016/j.immuni.2013.05.016, PMID: 23809161 PMC3730833

[ref67] Murakami T. Ockinger J. Yu J. Byles V. Mccoll A. Hofer A. M.. (2012). Critical role for calcium mobilization in activation of the NLRP3 inflammasome. Proc. Natl. Acad. Sci. U. S. A. 109, 11282–11287. doi: 10.1073/pnas.1117765109, PMID: 22733741 PMC3396518

[ref68] Nalawansha D. A. Crews C. M. (2020). Protacs: An emerging therapeutic modality in precision medicine. Cell Chem. Biol. 27, 998–1014. doi: 10.1016/j.chembiol.2020.07.020, PMID: 32795419 PMC9424844

[ref69] Ni P. Dong H. Zhou Q. Wang Y. Sun M. Qian Y.. (2019). Preoperative sleep disturbance exaggerates surgery-induced neuroinflammation and neuronal damage in aged mice. Mediat. Inflamm. 2019:8301725. doi: 10.1155/2019/8301725 PMC6442479 31011286

[ref70] Palazón-Riquelme P. Worboys J. D. Green J. Valera A. Martín-Sánchez F. Pellegrini C.. (2018). Usp7 and Usp47 deubiquitinases regulate NLRP3 inflammasome activation. EMBO Rep. 19:e44766. doi: 10.15252/embr.201744766, PMID: 30206189 PMC6172458

[ref71] Park Y. J. Yoon S. J. Suh H. W. Kim D. O. Park J. R. Jung H.. (2013). Txnip deficiency exacerbates endotoxic shock via the induction of excessive nitric oxide synthesis. PLoS Pathog. 9:e1003646. doi: 10.1371/journal.ppat.1003646, PMID: 24098117 PMC3789754

[ref72] Qin Y. Li Q. Liang W. Yan R. Tong L. Jia M.. (2021). Trim28 sumoylates and stabilizes NLRP3 to facilitate inflammasome activation. Nat. Commun. 12:4794. doi: 10.1038/s41467-021-25033-4, PMID: 34373456 PMC8352945

[ref73] Que Y. Y. Zhu T. Zhang F. X. Peng J. (2020). Neuroprotective effect of Dusp14 overexpression against isoflurane-induced inflammatory response, pyroptosis and cognitive impairment in aged rats through inhibiting the NLRP3 inflammasome. Eur. Rev. Med. Pharmacol. Sci. 24, 7101–7113. doi: 10.26355/eurrev_202006_21704, PMID: 32633405

[ref74] Rathkey J. K. Zhao J. Liu Z. Chen Y. Yang J. Kondolf H. C.. (2018). Chemical disruption of the pyroptotic pore-forming protein gasdermin D inhibits inflammatory cell death and sepsis. Sci. Immunol. 3:eaat2738. doi: 10.1126/sciimmunol.aat2738, PMID: 30143556 PMC6462819

[ref75] Rogers C. Fernandes-Alnemri T. Mayes L. Alnemri D. Cingolani G. Alnemri E. S. (2017). Cleavage of Dfna5 by caspase-3 during apoptosis mediates progression to secondary necrotic/pyroptotic cell death. Nat. Commun. 8:14128. doi: 10.1038/ncomms14128, PMID: 28045099 PMC5216131

[ref76] Safavynia S. A. Goldstein P. A. (2018). The role of neuroinflammation in postoperative cognitive dysfunction: moving from hypothesis to treatment. Front. Psych. 9:752. doi: 10.3389/fpsyt.2018.00752, PMID: 30705643 PMC6345198

[ref77] Schorn C. Frey B. Lauber K. Janko C. Strysio M. Keppeler H.. (2011). Sodium overload and water influx activate the Nalp3 inflammasome. J. Biol. Chem. 286, 35–41. doi: 10.1074/jbc.M110.139048 21051542 PMC3012992

[ref78] Schrauben M. Dempster E. Lunnon K. (2020). Applying gene-editing technology to elucidate the functional consequence of genetic and epigenetic variation in Alzheimer's disease 30, 992–1004. doi: 10.1111/bpa.12881, PMID: 32654206 PMC8018012

[ref79] Shao A. Fei J. Feng S. Weng J. (2020). Chikusetsu saponin Iva alleviated sevoflurane-induced neuroinflammation and cognitive impairment by blocking NLRP3/caspase-1 pathway. Pharmacol. Rep. 72, 833–845. doi: 10.1007/s43440-020-00078-2 32124392

[ref80] Shao L. Liu Y. Wang W. Li A. Wan P. Liu W.. (2020). Sumo1 sumoylates and Senp3 desumoylates NLRP3 to orchestrate the inflammasome activation. FASEB J. 34, 1497–1515. doi: 10.1096/fj.201901653R 31914638

[ref81] Shi J. Gao W. Shao F. (2017). Pyroptosis: gasdermin-mediated programmed necrotic cell death. Trends Biochem. Sci. 42, 245–254. doi: 10.1016/j.tibs.2016.10.004 27932073

[ref82] Shi J. Zhao Y. Wang Y. Gao W. Ding J. Li P.. (2014). Inflammatory caspases are innate immune receptors for intracellular LPS. Nature 514, 187–192. doi: 10.1038/nature13683, PMID: 25119034

[ref83] Shi J. Zhao Y. Wang K. Shi X. Wang Y. Huang H.. (2015). Cleavage of Gsdmd by inflammatory caspases determines pyroptotic cell death. Nature 526, 660–665. doi: 10.1038/nature15514, PMID: 26375003

[ref84] Shim D. W. Shin W. Y. Yu S. H. Kim B. H. Ye S. K. Koppula S.. (2017). Bot-4-one attenuates NLRP3 inflammasome activation: NLRP3 alkylation leading to the regulation of its ATPase activity and ubiquitination. Sci. Rep. 7:15020. doi: 10.1038/s41598-017-15314-8, PMID: 29118366 PMC5678161

[ref85] Silva M. C. Ferguson F. M. (2019). Targeted degradation of aberrant tau in frontotemporal dementia patient-derived neuronal cell models. Elife 8:e45457. doi: 10.7554/eLife.45457, PMID: 30907729 PMC6450673

[ref86] Song N. Liu Z. S. Xue W. Bai Z. F. Wang Q. Y. Dai J.. (2017). NLRP3 phosphorylation is an essential priming event for inflammasome activation. Mol. Cell 68, 185–197.e6. doi: 10.1016/j.molcel.2017.08.017, PMID: 28943315

[ref87] Song P. Yi Z. Fu Y. Song D. Chen K. Zheng J.. (2021). Reversing postcardiopulmonary bypass associated cognitive dysfunction using k-opioid receptor agonists to regulate microglial polarization via the NLRP3/Caspase-1 pathway. J. Healthc. Eng. 2021:3048383. doi: 10.1155/2021/3048383, PMID: 34630980 PMC8500742

[ref88] Soriano-Teruel P. M. García-Laínez G. Marco-Salvador M. Pardo J. Arias M. Deford C.. (2021). Identification of an ASC oligomerization inhibitor for the treatment of inflammatory diseases. Cell Death Dis. 12:1155. doi: 10.1038/s41419-021-04420-1, PMID: 34903717 PMC8667020

[ref89] Sridhar R. Lakshminarayanan R. Madhaiyan K. Amutha Barathi V. Lim K. H. C. Ramakrishna S. (2015). Electrosprayed nanoparticles and electrospun nanofibers based on natural materials: applications in tissue regeneration, drug delivery and pharmaceuticals. Chem. Soc. Rev. 44, 790–814. doi: 10.1039/c4cs00226a, PMID: 25408245

[ref90] Swanson K. V. Deng M. Ting J. P. (2019). The NLRP3 inflammasome: molecular activation and regulation to therapeutics. Nat. Rev. Immunol. 19, 477–489. doi: 10.1038/s41577-019-0165-0, PMID: 31036962 PMC7807242

[ref91] Tabrizi S. J. Flower M. D. Ross C. A. Wild E. J. (2020). Huntington disease: new insights into molecular pathogenesis and therapeutic opportunities. Nat. Rev. Neurol. 16, 529–546. doi: 10.1038/s41582-020-0389-4 32796930

[ref92] Tang T. Lang X. Xu C. Wang X. Gong T. Yang Y.. (2017). CLICs-dependent chloride efflux is an essential and proximal upstream event for NLRP3 inflammasome activation. Nat. Commun. 8:202. doi: 10.1038/s41467-017-00227-x, PMID: 28779175 PMC5544706

[ref93] Tang T. Li P. Zhou X. Wang R. Fan X. Yang M.. (2021). The E3 ubiquitin ligase TRIM65 negatively regulates inflammasome activation through promoting ubiquitination of NLRP3. Front. Immunol. 12:741839. doi: 10.3389/fimmu.2021.741839, PMID: 34512673 PMC8427430

[ref94] Tang J. Xiao Y. Lin G. Guo H. Deng H. X. Tu S.. (2021). Tyrosine phosphorylation of NLRP3 by the Src family kinase Lyn suppresses the activity of the NLRP3 inflammasome. Sci. Signal. 14:eabe3410. doi: 10.1126/scisignal.abe3410, PMID: 34699250 PMC8815314

[ref95] Terrando N. Eriksson L. I. Ryu J. K. Yang T. Monaco C. Feldmann M.. (2011). Resolving postoperative neuroinflammation and cognitive decline. Ann. Neurol. 70, 986–995. doi: 10.1002/ana.22664, PMID: 22190370 PMC4556354

[ref96] Vanderweyde T. Bednar M. M. Forman S. A. Wolozin B. (2010). Iatrogenic risk factors for Alzheimer's disease: surgery and anesthesia. J. Alzheimers Dis. 22, 91–104. doi: 10.3233/jad-2010-100843, PMID: 20858967 PMC3108154

[ref97] Wan P. Zhang Q. Liu W. Jia Y. Ai S. Wang T.. (2019). Cullin1 binds and promotes NLRP3 ubiquitination to repress systematic inflammasome activation. FASEB J. 33, 5793–5807. doi: 10.1096/fj.201801681R, PMID: 30653357

[ref98] Wang Y. Gao W. Shi X. Ding J. Liu W. He H.. (2017). Chemotherapy drugs induce pyroptosis through caspase-3 cleavage of a gasdermin. Nature 547, 99–103. doi: 10.1038/nature22393, PMID: 28459430

[ref99] Wang L. Liu H. Zhang L. Wang G. Zhang M. Yu Y. (2017). Neuroprotection of dexmedetomidine against cerebral ischemia-reperfusion injury in rats: involved in inhibition of Nf-κB and inflammation response. Biomol. Ther. 25, 383–389. doi: 10.4062/biomolther.2015.180, PMID: 27871154 PMC5499616

[ref100] Wang Z. Meng S. Cao L. Chen Y. Zuo Z. Peng S. (2018). Critical role of NLRP3-caspase-1 pathway in age-dependent isoflurane-induced microglial inflammatory response and cognitive impairment. J. Neuroinflammat. 15:109. doi: 10.1186/s12974-018-1137-1 PMC5904978 29665808

[ref101] Wang T. Ruan B. Wang J. Zhou Z. Zhang X. Zhang C.. (2022). Activation of NLRP3-Caspase-1 pathway contributes to age-related impairments in cognitive function and synaptic plasticity. Neurochem. Int. 152:105220. doi: 10.1016/j.neuint.2021.105220, PMID: 34743016

[ref102] Wei P. Yang F. Zheng Q. Tang W. Li J. (2019). The potential role of the NLRP3 inflammasome activation as a link between mitochondria ROS generation and neuroinflammation in postoperative cognitive dysfunction. Front. Cell. Neurosci. 13:73. doi: 10.3389/fncel.2019.00073, PMID: 30873011 PMC6401615

[ref103] Wen-Yuan W. Wan-Qing Y. Qi-Yun H. Yu-Si L. Shao-Jie Q. Jin-Tao L.. (2022). mTORC1-dependent and GSDMD-mediated pyroptosis in developmental sevoflurane neurotoxicity. Mol. Neurobiol. doi: 10.1007/s12035-022-03070-4. [Epub ahead of print], PMID: 36224321

[ref104] Wu Z. Tan J. Lin L. Zhang W. Yuan W. (2022). microRNA-140-3p protects hippocampal neuron against pyroptosis to attenuate sevoflurane inhalation-induced post-operative cognitive dysfunction in rats via activation of Htr2A/Erk/Nrf2 axis by targeting DNMT1. Cell Death Discov. 8:290. doi: 10.1038/s41420-022-01068-4, PMID: 35710537 PMC9203584

[ref105] Xia L. Liu L. Wang Q. Ding J. Wang X. (2021). Relationship between the pyroptosis pathway and epilepsy: A bioinformatic analysis. Front. Neurol. 12:782739. doi: 10.3389/fneur.2021.782739, PMID: 35095728 PMC8795950

[ref106] Xiang X. Yu Y. Tang X. Chen M. Zheng Y. Zhu S. (2019). Transcriptome profile in hippocampus during acute inflammatory response to surgery: toward early stage of PND. Front. Immunol. 10:149. doi: 10.3389/fimmu.2019.00149, PMID: 30804943 PMC6370675

[ref107] Xiao S. Zhou D. Luan P. Gu B. Feng L. Fan S.. (2016). Graphene quantum dots conjugated neuroprotective peptide improve learning and memory capability. Biomaterials 106, 98–110. doi: 10.1016/j.biomaterials.2016.08.021, PMID: 27552320

[ref108] Xing Y. Yao X. Li H. Xue G. Guo Q. Yang G.. (2017). Cutting edge: Traf6 mediates TLR/Il-1R signaling-induced nontranscriptional priming of the NLRP3 inflammasome. J. Immunol. 199, 1561–1566. doi: 10.4049/jimmunol.1700175, PMID: 28739881

[ref109] XuG.LuH.DongY.ShapovalD.SorianoS. G.LiuX.. (2017). Coenzyme Q10 reduces sevoflurane-induced cognitive deficiency in young mice. Br. J. Anaesth. 119, 481–491. doi: 10.1093/bja/aex071, PMID: 28482003

[ref110] Yamashita H. Tomoshige S. Nomura S. Ohgane K. Hashimoto Y. Ishikawa M. (2020). Application of protein knockdown strategy targeting β-sheet structure to multiple disease-associated polyglutamine proteins. Bioorg. Med. Chem. 28:115175. doi: 10.1016/j.bmc.2019.115175, PMID: 31767406

[ref111] Yang T. Feng X. Zhao Y. Zhang H. Cui H. Wei M.. (2020). Dexmedetomidine enhances autophagy via α2-AR/AMPK/mTOR pathway to inhibit the activation of NLRP3 inflammasome and subsequently alleviates lipopolysaccharide-induced acute kidney injury. Front. Pharmacol. 11:790. doi: 10.3389/fphar.2020.00790, PMID: 32670056 PMC7326938

[ref112] Yao K. Mu Q. Zhang Y. Cheng Q. Cheng X. Liu X.. (2022). Hesperetin nanoparticle targeting neutrophils for enhanced TBI therapy. Adv. Funct. Mater. 32:2205787. doi: 10.1002/adfm.202205787

[ref113] Yin L. Bao F. Wu J. Li K. (2018). NLRP3 inflammasome-dependent pyroptosis is proposed to be involved in the mechanism of age-dependent isoflurane-induced cognitive impairment. J. Neuroinflammat. 15:266. doi: 10.1186/s12974-018-1299-x, PMID: 30217191 PMC6138927

[ref114] Yu Y. Yang Y. Tan H. Boukhali M. Khatri A. Yu Y.. (2020). Tau contributes to sevoflurane-induced neurocognitive impairment in neonatal mice. Anesthesiology 133, 595–610. doi: 10.1097/aln.0000000000003452 32701572 PMC7429299

[ref115] Zewinger S. Reiser J. Jankowski V. Alansary D. Hahm E. Triem S.. (2020). Apolipoprotein C3 induces inflammation and organ damage by alternative inflammasome activation. Nat. Immunol. 21, 30–41. doi: 10.1038/s41590-019-0548-1, PMID: 31819254

[ref116] Zhang Z. Bai H. Ma X. Shen M. Li R. Qiu D.. (2021). Blockade of the NLRP3/caspase-1 axis attenuates ketamine-induced hippocampus pyroptosis and cognitive impairment in neonatal rats. J. Neuroinflammation 18:239. doi: 10.1186/s12974-021-02295-9, PMID: 34666787 PMC8527745

[ref117] Zhang L. Ko C. J. Li Y. Jie Z. Zhu L. Zhou X.. (2021a). Peli1 facilitates NLRP3 inflammasome activation by mediating ASC ubiquitination. Cell Rep. 37:109904. doi: 10.1016/j.celrep.2021.109904 34706239 PMC12011377

[ref118] Zhang Z. Ma Q. Velagapudi R. Barclay W. E. Rodriguiz R. M. Wetsel W. C.. (2022). Annexin-A1 Tripeptide attenuates surgery-induced neuroinflammation and memory deficits through regulation the NLRP3 inflammasome. Front. Immunol. 13:856254. doi: 10.3389/fimmu.2022.856254, PMID: 35603196 PMC9120413

[ref119] Zhang H. Qin C. An C. Zheng X. Wen S. Chen W.. (2021). Application of the Crispr/Cas9-based gene editing technique in basic research, diagnosis, and therapy of cancer. Mol. Cancer 20:126. doi: 10.1186/s12943-021-01431-6, PMID: 34598686 PMC8484294

[ref120] Zhang L. Xiao F. Zhang J. Wang X. Ying J. Wei G.. (2021b). Dexmedetomidine mitigated NLRP3-mediated neuroinflammation via the ubiquitin-autophagy pathway to improve perioperative neurocognitive disorder in mice. Front. Pharmacol. 12:646265. doi: 10.3389/fphar.2021.646265, PMID: 34079457 PMC8165564

[ref121] Zhang X. Zhang Y. Li R. Zhu L. Fu B. Zyan T. (2020). Salidroside ameliorates Parkinson's disease by inhibiting NLRP3-dependent pyroptosis. Aging (Albany NY) 12, 9405–9426. doi: 10.18632/aging.103215, PMID: 32432571 PMC7288953

[ref122] Zhang J. Zhu S. Jin P. Huang Y. Dai Q. Zhu Q.. (2020). Graphene oxide improves postoperative cognitive dysfunction by maximally alleviating amyloid beta burden in mice. Theranostics 10, 11908–11920. doi: 10.7150/thno.50616, PMID: 33204319 PMC7667672

[ref123] Zhong Z. Liang S. Sanchez-Lopez E. He F. Shalapour S. Lin X. J.. (2018). New mitochondrial DNA synthesis enables NLRP3 inflammasome activation. Nature 560, 198–203. doi: 10.1038/s41586-018-0372-z 30046112 PMC6329306

[ref124] Zhou Z. He H. Wang K. Shi X. Wang Y. Su Y.. (2020). Granzyme A from cytotoxic lymphocytes cleaves GSDMB to trigger pyroptosis in target cells. Science 368:eaaz7548. doi: 10.1126/science.aaz7548, PMID: 32299851

[ref125] Zhou R. Yazdi A. S. Menu P. Tschopp J. (2011). A role for mitochondria in NLRP3 inflammasome activation. Nature 469, 221–225. doi: 10.1038/nature09663 21124315

[ref126] Zuo Y. Yin L. Cheng X. Li J. Wu H. Liu X.. (2020). Elamipretide attenuates pyroptosis and perioperative neurocognitive disorders in aged mice. Front. Cell. Neurosci. 14:251. doi: 10.3389/fncel.2020.00251, PMID: 32903868 PMC7439217

